# The association between total bile acid and bone mineral density among patients with type 2 diabetes

**DOI:** 10.3389/fendo.2023.1153205

**Published:** 2023-03-24

**Authors:** Song Yang, Hongyun Li, Yuanyuan Gu, Qiang Wang, Li Dong, Chao Xu, Yuxin Fan, Ming Liu, Qingbo Guan, Lixing Ma

**Affiliations:** ^1^ Department of Endocrinology, Tianjin Medical University General Hospital, Tianjin, China; ^2^ Department of Endocrinology, The Affiliated Taian City Central Hospital of Qingdao University, Taian, China; ^3^ School of Public Health, Shandong First Medical University and Shandong Academy of Medical Sciences, Jinan, China; ^4^ Department of Pharmacy, The Affiliated Taian City Central Hospital of Qingdao University, Taian, China; ^5^ Department of Joint Surgery, The Affiliated Taian City Central Hospital of Qingdao University, Taian, China; ^6^ School of Public Health, Cheeloo College of Medicine, Shandong University, Jinan, China; ^7^ Department of Endocrinology, Shandong Provincial Hospital Affiliated to Shandong First Medical University, Jinan, China; ^8^ Department of Gastroenterology, The Second Affiliated Hospital of Shandong First Medical University, Taian, China

**Keywords:** type 2 diabetes mellitus (T2DM), total bile acid (TBA), bone mineral density (BMD), osteoporosis, abnormal bone metabolism

## Abstract

**Objective:**

Bile acids have underlying protective effects on bones structure. Long-term diabetes also causes skeletal disorders including osteoporosis, Charcot arthropathy and renal osteodystrophy. Nevertheless, few studies have reported whether bile acid is associated with bone metabolism in diabetics. This study aimed to explore the relationship between total bile acid (TBA) and bone mineral density (BMD) among patients with type 2 diabetes mellitus (T2DM).

**Methods:**

We retrospectively included 1,701 T2DM patients who were hospitalized in Taian City Central Hospital (TCCH), Shandong Province, China between January 2017 to December 2019. The participants were classified into the osteopenia (n = 573), osteoporosis (n= 331) and control groups (n= 797) according to BMD in the lumbar spine and femoral. The clinical parameters, including TBA, bilirubin, vitamin D, calcium, phosphorus and alkaline phosphatase were compared between groups. Multiple linear regression was used to analyze the relationship between TBA and BMD in lumbar spine, femoral, trochiter, ward’s triangle region. A logistic regression was conducted to develop a TBA-based diagnostic model for differentiating abnormal bone metabolism from those with normal BMD. We evaluated the performance of model using ROC curves.

**Results:**

The TBA level was significantly higher in patients with osteoporosis (Median[M]= 3.300 μmol/L, interquartile range [IQR] = 1.725 to 5.250 μmol/L) compared to the osteopenia group (M = 3.200 μmol/L, IQR = 2.100 to 5.400 μmol/L) and control group (M = 2.750 μmol/L, IQR = 1.800 to 4.600 μmol/L) (*P <*0.05). Overall and subgroup analyses indicated that TBA was negatively associated with BMD after adjusted for the co-variates (i.e., age, gender, diabetes duration, BMI, total bilirubin, direct bilirubin, indirect bilirubin) (*P <*0.05). Logistic regression revealed that higher TBA level was associated with increased risk for abnormal bone metabolism (OR = 1.044, 95% CI = 1.005 to 1.083). A TBA-based diagnostic model was established to identify individuals with abnormal bone metabolism (T-score ≤ -1.0). The area under ROC curve (AUC) of 0.767 (95% CI = 0.730 to 0.804).

**Conclusion:**

Our findings demonstrated the potential role of bile acids in bone metabolism among T2DM patients. The circulating TBA might be employed as an indicator of abnormal bone metabolism.

## Introduction

Type 2 diabetes mellitus (T2DM), one of metabolic diseases, is mainly caused by insulin deficiency or resistance (1). More than 460 million persons suffer from T2DM globally, accounting for 6.28% of the world’s population in 2020 (2). Long-term diabetes commonly induces dysfunctions in multiple tissues and organs, such as brain, cardiovascular system, kidneys and eyes (3). Besides, skeletal disorders have been observed in association with DM, including osteoporosis, Charcot arthropathy and renal osteodystrophy (4). It is believed that disorders of glucose metabolism can damage bone microstructure and increase the incidences of osteoporosis and osteoporosis-associated fracture (5, 6). Bone mineral density (BMD) is a key parameter of bone health and an osteoporosis predictor (7, 8). Clinical evidences have evidenced that T2DM increases the risk of low BMD, osteoporosis and fractures, particularly in older men and postmenopausal women (9).

Total bile acids (TBA), a series of signaling molecules synthesized by liver cells, display biological functions, such as metabolism of glucose and lipid, and regulation of intestinal flora (10). Bile acid-induced activation of G protein-coupled bile acid receptor (TGR5) promotes insulin secretion by increasing intracellular calcium concentration (11). Studies have also identified that circulating TBA was positively correlated with BMD, indicating the potential role of bile acids in the regulation of bone metabolism (12, 13). Bile acids regulate bone metabolism *via* the activation of nuclear receptor, farnesoid X receptor (FXR), membrane receptor, TGR5 and intestinal flora (14–16).

Since TBA regulating bone metabolism is one of the pathophysiological pathways of osteoporosis, we hypothesized that TBA is associated with osteoporosis in diabetic. However, to date, no studies have reported the association with TBA and BMD in diabetic patients. We conducted this retrospective study to identify the relationship between serum TBA and bone metabolism, and to explore the potential role of TBA in the development of osteoporosis in diabetics.

## Methods

### Study participants

A total of 550 T2DM patients who did not fulfill the inclusion criteria or lacked clinical data were excluded. Finally, 1701 patients with T2DM were included from Taian City Central Hospital (TCCH) between January 2017 and December 2019 (**Figure S1**). The participants were classified into three groups: (1) osteoporosis, (2) osteopenia, and (3) control groups.

The diagnosis of T2DM and osteoporosis was based on World Health Organization (WHO) criteria (17–19), T-score ≤ -2.5 for osteoporosis, between -2.5 to -1.0 for osteopenia, > -1.0 for normality and T-score ≤ -1.0 for abnormal bone metabolism.

Inclusion criteria were as follows: (1) Individuals diagnosed with T2DM; (2) No severe somatic disorders including cardiovascular diseases and cancers; (3) No mental disorders; (4) No diabetic acute complications, including ketoacidosis, lactic acidosis and diabetic hyperosmolarity; (5) Not taking any medications that affect bone metabolism and bile acid metabolism in 6 months. Exclusion criteria: (1) Patients diagnosed with T1DM, gestational diabetes mellitus or other specific types of diabetes; (2) Patients with chronic kidney insufficiency, chronic hepatic insufficiency, liver or renal dysfunction; (3) Patients with endocrine diseases that affect bone metabolism, including parathyroid dysfunction, gonadal diseases and adrenal diseases; (4) Patients with diseases that seriously affect bone metabolism and lead to secondary osteoporosis, such as rheumatic diseases, hematological diseases and digestive disease; (5) Individuals with family history of osteoporosis; (6) Patients with a history of recent exposure to radioactive materials; (7) Patients with history of prolonged bed rest.

This study has been reviewed and approved by the ethics committee of TCCH (No. 2021-05-001). As a retrospective study of clinical dataset, this research was exempt from the request of informed consent from subjects.

### Data collection

The characteristics of age, gender, height, weight, body mass index (BMI), systolic blood pressure (SBP), diastolic blood pressure (DBP) and diabetes duration were collected from clinical records.

Total cholesterol (TC), triglyceride (TG), low density lipoprotein cholesterol (LDL), high density lipoprotein cholesterol (HDL), calcium ions, phosphorus, alkaline phosphatase (ALP), TBA, total bilirubin, direct bilirubin and indirect bilirubin were measured by Modular P800 automatic biochemical analyzer (Roche, German). Glycated hemoglobin A1c (HbA1c) was detected *via* high-performance liquid chromatography (Bio-Rad Laboratories, CA, USA). Fasting blood glucose (FBG) was measured with an automatic analyzer (Hitachi, Tokyo, Japan). Fins, C-peptide and vitamin D were determined by Cobas 6000 electro chemiluminescence (Roche, German). BMD of lumbar spine, femoral, trochiter and ward’s triangle region were measured by dual energy X-ray absorptiometry (GE Lunar IDXA, USA).

### Statistical analysis

Continuous data were presented as means and standard deviations (SDs) when normally distributed, otherwise presented as median (M) and interquartile range (IQR). Categorical data were presented as frequencies. For comparisons between multiple groups, one-way analysis of variance (ANOVA) followed by Least-Significant Difference (LSD) test was used for normally distributed data. Kruskal-Wallis test followed by Bonferroni *post hoc* test was used for non-normal distributed data. Chi-square test was used for comparison of categorical data. Jonckheere–Terpstra test was used to assess the trend in TBA level between multiple groups. Multiple linear regression was used to analyze the associations between TBA and BMD. Logistic regression analysis was used to establish a TBA-based diagnostic model to identify individuals with abnormal bone metabolism from those with normal (T-score ≤ -1.0). The receiver operator characteristic (ROC) curve and the area under the curve (AUC) were employed to evaluate the model’s performance. Subgroup analysis is performed to assess the association between TBA and BMD based on gender, age group, BMI and menstrual conditions.

A two-side *P*-value < 0.05 was considered statistically significant. Statistical analyses were performed using R packages 4.1.0 (R Core Team) and SPSS 25.0 (IBM, New York).

## Results

### Clinical characteristics of the participants

**Figure 1:**
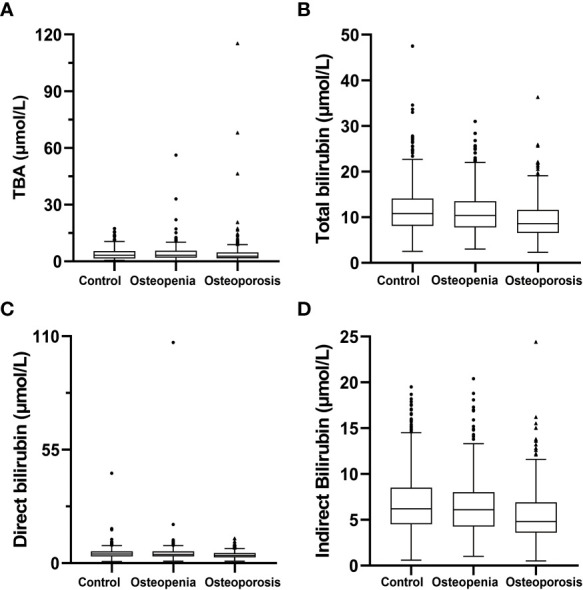
Hepatobiliary metabolism indicators. **(A)** total bile acid; **(B)** total bilirubin; **(C)** direct bilirubin; **(D)** indirect bilirubin.

The basic characteristics of the 1,701 T2DM patients are listed in **Table 1**. They were classified as control group: 797 individuals with normal BMD, aged (54.8 ± 11.3) years, of whom 68.4% (545/797) were male; osteopenia group: 573 individuals with osteopenia, aged (61.9 ± 9.2) years, of whom 50.3% (288/573) were male; and osteoporosis group: 331 individuals with osteoporosis, aged (67.1 ± 8.7) years, of whom 21.1% (70/331) were male. The results of hepatobiliary metabolism indicators are shown in **Table 2** and **Figure 1**. The results of bone metabolism indicators are presented in **Table 3**.

**Table 1:** Characteristic description of T2DM patients.

Indicators	Osteoporosis (n=331)	Osteopenia (n=573)	Control (n=797)	χ²/*F*	*P*
Male [n (%)]	70 (21.1)	288 (50.3)	545 (68.4)	212.273	<0.001
Female [n (%)]	261 (78.9)	285 (49.7)	252 (31.6)		
Age (year)	67.1 ± 8.7 ^*#^	61.9 ± 9.2^#^	54.8 ± 11.3	195.389	<0.001
Diabetes duration (month)	120.0 (72.0,204.0) ^*#^	120.0 (48.0, 180.0) ^#^	84.0 (36.0, 144.0)	46.854	<0.001
BMI<25 [n (%)]	195 (85.5)	269 (47.7)	304 (38.8)	41.020	<0.001
BMI≥25 [n (%)]	33 (14.5)	295 (52.3)	480 (61.2)		
SBP	143 ± 19^#^	141 ± 21^#^	139 ± 20	4.665	0.010
DBP	76 ± 11^*#^	78 ± 11^#^	81 ± 11	22.305	0.001
TC (mmol/L)	4.540 (3.655,5.365)	4.460 (3.600, 5.295)	4.510 (3.700,5.300)	0.905	0.636
TG (mmol/L)	1.310 (0.890,2.075) ^#^	1.380 (0.960,2.125)	1.470 (1.010, 2.412)	7.570	0.023
LDL (mmol/L)	2.780 (2.110,3.483)	2.800 (2.010,3.390)	2.870 (2.170,3.500)	1.641	0.440
HDL (mmol/L)	1.370 (1.160,1.670) ^#^	1.320 (1.100,1.161)	1.270 (1.080,1.510)	17.978	<0.001
C-Peptide (ng/ml)	0.910 (0.600,1.520) ^#^	1.050 (0.640,1.600)	1.150 (0.708,1.760)	8.265	0.016
FINS (uIU/ml)	3.300 (1.725,5.250)	8.145 (5.538,12.335)	8.750 (6.100,13.523)	3.773	0.152
HbA1C (%)	8.786 ± 2.366	8.945 ± 2.257	8.957 ± 2.188	0.639	0.528
FBG (mmol/L)	9.250 (6.980,12.060)^#^	9.235 (7.355,12.260)	9.820 (7.630,13.185)	11.942	0.003

BMI, body mass index; SBP, Systolic blood pressure; DBP, diastolic blood pressure; FINS, Fasting insulin; HbA1c, Hemoglobin A1c; HDL-C, high-density lipoprotein cholesterol; LDL-C, low-density lipoprotein cholesterol; TC, cholesterol; TG, triglycerides; FBG, Fasting blood glucose; ^*^ *P* < 0.05 compared with osteopenia group; ^#^ *P* < 0.05 compared with the controls group.

**Table 2:** Hepatobiliary metabolism indicators of T2DM patients.

Indicators	Osteoporosis (n=331)	Osteopenia (n=573)	Control (n=797)	χ²	*P*
TBA (μmol/L)	3.300 (1.725,5.250)	3.200 (2.100,5.400) ^#^	2.750 (1.800,4.600)	6.435	0.040
Total bilirubin (μmol/L)	8.600 (6.650,11.600) ^*#^	10.400 (7.775,13.500)	10.800 (8.100,14.100)	48.848	<0.001
Direct bilirubin (μmol/L)	3.700 (3.000,4.750) ^*#^	4.300 (3.400,5.500)	4.400 (3.400,5.500)	35.291	<0.001
Indirect Bilirubin (μmol/L)	4.800 (3.600,6.900) ^*#^	6.100 (4.250,8.000)	6.200 (4.500,8.500)	40.169	<0.001

TBA, total bile acid; ^*^ *P* < 0.05 compared with osteopenia group; ^#^ *P* < 0.05 compared with the controls group.

**Table 3:** Bone metabolism indicators of T2DM patients.

Indicators	Osteoporosis (n=331)	Osteopenia (n=573)	Control (n=797)	χ²/*F*	*P*
Calcium ion (mmol/L)	2. 380 (2.300, 2.460)	2.380 (2.310, 2.445)	2.380 (2.310, 2.450)	1.222	0.295
Phosphorus (mmol/L)	1.170 (1.065,1.280)	1.180 (1.030, 1.280)	1.170 (1.043, 1.290)	0.608	0.738
Vitamin D (ng/ml)	17.150 (11.800,22.275)	17.750 (12.600, 24.200)	18.600 (13.750, 23.775)	4.325	0.115
ALP (u/l)	71.00 (61.00,87.00) ^#^	71.00 (60.00, 85.25) ^#^	67.00 (56.00, 82.00)	4.664	0.010
L1BMD (g/cm²)	0.787 ± 0.167^*^#^^	0.940 ± 0.150^#^	1.100 ± 0.177	433.627	<0.001
L2BMD (g/cm²)	0.831 ± 0.135^*^#^^	1.033 ± 0.150^#^	1.203 ± 0.171	662.445	<0.001
L3BMD (g/cm²)	0.911 ± 0.158^*^#^^	1.124 ± 0.162^#^	1.286 ± 0.186	554.659	<0.001
L4BMD (g/cm²)	0.942 ± 0.172^*^#^^	1.135 ± 0.176^#^	1.281 ± 0.200	389.901	<0.001
Total lumbar spine BMD (g/cm²)	0.872 ± 0.133^*^#^^	1.068 ± 0.147^#^	1.228 ± 0.176	590.632	<0.001
Femoral neck BMD (g/cm²)	0.726 ± 0.309^*^#^^	0.821 ± 0.128^#^	1.001 ± 0.256	190.556	<0.001
Trochiter BMD (g/cm²)	0.635 ± 0.113^*^#^^	0.749 ± 0.133^#^	0.881 ± 0.147	411.295	<0.001
Femoral shaft BMD (g/cm²)	0.967 ± 0.194^*^#^^	1.133 ± 0.198^#^	1.288 ± 0.185	339.836	<0.001
Ward’s triangle region BMD (g/cm²)	0.514 ± 0.122^*^#^^	0.636 ± 0.115^#^	0.800 ± 0.290	109.966	<0.001
Total femoral BMD (g/cm²)	0.827 ± 0.521^*^#^^	0.916 ± 0.139^#^	1.076 ± 0.197	25.255	<0.001

ALP, Alkaline Phosphatase; L1BMD, first lumbar vertebra BMD; L2BMD, second lumbar vertebra BMD; L3 BMD, third lumbar vertebra BMD; L4 BMD, fourth lumbar vertebra BMD; ^*^ *P* < 0.05 compared with osteopenia group; ^#^ *P* < 0.05 compared with the controls group.

Significant differences were identified between age, gender, BMI, diabetes duration, total bilirubin, direct bilirubin, indirect bilirubin, SBP, DBP, FBG, TG, LDL, C-peptide, ALP and BMD indicators between the multigroup (i.e., control, osteopenia and osteoporosis groups) (*P* < 0.05).

Age showed a gradual increase in the values and there was a significant difference in pairwise comparisons between the multigroup. In addition, DBP presented a decreasing trend among control, osteopenia and osteoporosis groups (*P* < 0.05) (**Table 1**).

### The relevance between serum TBA levels and BMD

The serum TBA levels in the osteopenia group were (3.200 μmol/L [IQR = 2.100 to 5.400 μmol/L]), which were significantly higher than that in the control group (2.750 μmol/L, [IQR = 1.800 to 4.600 μmol/L]) (P < 0.05). Furthermore, Jonckheere–Terpstra test found TBA levels presented a significant increasing trend among control, osteopenia and osteoporosis groups (*P*< 0.05) (**Table 2**).

Multiple linear regression revealed that TBA level were independent determinants associated with BMD in third lumbar vertebrae (L3), fourth lumbar vertebrae (L4), total lumbar spine, femoral neck, and ward’s triangle region (*P*< 0.05) (**Table 4**).

**Table 4:** The association of TBA levels with BMD.

Sites	*β*	95% CI of *β*	*P*
L3 BMD	-0.003	-0.003 ~ -0.001	0.019
L4 BMD	-0.003	-0.005 ~ -0.001	0.043
Total lumbar spine BMD	-0.003	-0.005 ~ -0.001	0.013
Femoral neck BMD	-0.003	-0.004 ~ -0.001	0.037
Ward’s triangle region BMD	-0.004	-0.007 ~ -0.001	0.003

BMD, bone mineral density; TBA, total bile acid; *β*, regression coefficient; CI, confidence intervals; L3, third lumbar vertebra; L4, fourth lumbar vertebra; Adjusted for gender, age, BMI, diabetes duration, total bilirubin, direct bilirubin, and indirect bilirubin.

### Subgroup analysis

Based on gender, age group (classify into < 60 and ≥ 60 years), BMI (classify into < 25 and ≥ 25) and menstrual conditions, a subgroup analysis is listed in **Tables S1**–**S3**.

Among the participants aged < 60 and BMI < 25, including men and women, TBA level was negatively associated with total lumbar spine BMD (**Table S1**). For men with normal BMI (BMI<25), TBA level was negatively associated with BMD levels in the femoral and ward’s triangle region (**Table S2**). In postmenopausal population, TBA level was negatively associated with the BMD levels in first lumbar vertebrae(L1), second lumbar vertebrae(L2), third lumbar vertebrae (L3), fourth lumbar vertebrae (L4), femoral neck and total lumbar spine (**Table S3**).

### TBA-based diagnostic model for abnormal bone metabolism

**Figure 2:**
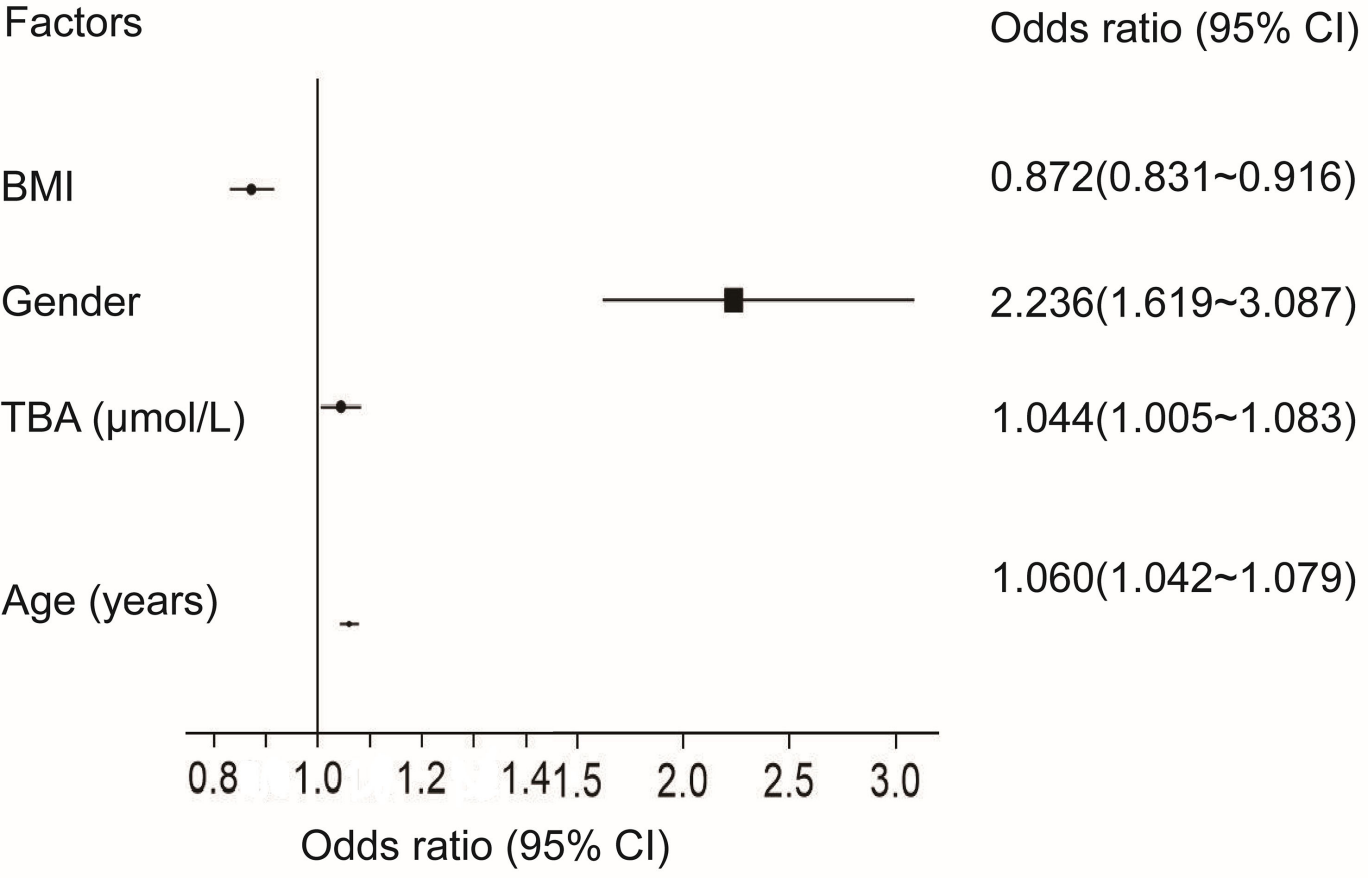
Forest plots of logistic regression analysis. CI, confidence interval.

Logistic regression analysis was used to establish a TBA-based diagnostic model to identify individuals with abnormal bone metabolism among T2DM patients (**Table 5**). A higher TBA level (OR = 1.044, 95% CI = 1.005 to 1.083) was associated with increased risk for abnormal bone metabolism. Older age (OR = 1.060, 95% CI = 1.042 to 1.079) and female (OR = 2.236, 95% CI = 1.619 to 3.087) correlated with a higher risk for abnormal bone metabolism. Higher BMI (OR =0.872, 95% CI =0.831 to 0.916) was associated with a lower risk for abnormal bone metabolism (**Figure 2**).

**Table 5:** Logistic regression of abnormal bone metabolism influence factors in T2DM patients.

Factors	*β*	*SE*	Walds χ2	*P*	OR (95%CI)
Gender (male)	0.805	0.165	23.909	<0.001	2.236 (1.619~3.087)
Age	0.059	0.009	42.355	<0.001	1.060 (1.042~1.079)
Diabetes duration	0.001	0.001	2.051	0.152	1.001 (0.999~1.003)
BMI	-0.136	0.025	30.765	<0.001	0.872 (0.831~0.916)
TBA	0.043	0.019	5.017	0.025	1.044 (1.005~1.083)

BMI, body mass index; TBA, total bile acid; SE, standard error; OR, odds ratio; CI, confidence intervals; *β*, regression coefficient.

**Figure 3:**
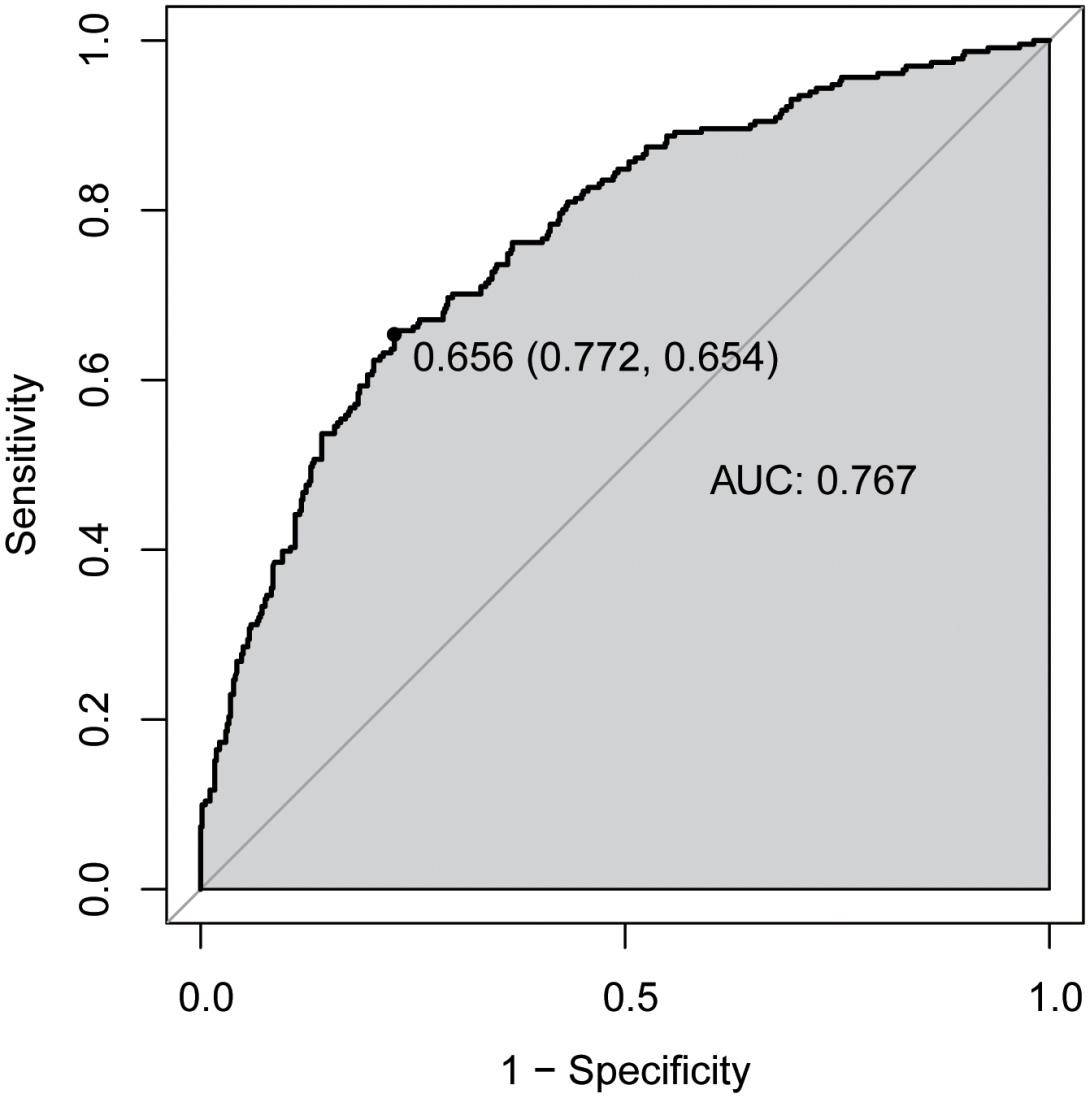
Receiver operator characteristic (ROC) curves for diagnostic model of abnormal bone metabolism. AUC, area under the curve.

We then established a diagnostic model using TBA, age, gender and BMI. As depicted in **Figure 3**, a ROC curve was used to assess the performance of model, which showed the AUC of 0.767(95% CI = 0.730 to 0.804), with a sensitivity of 65.4% and a specificity of 77.2% at a cut-off-value of 0.656.

## Discussion

Our findings identify, for the first time in T2DM patients, the TBA level among diabetic patients with osteoporosis was higher than those with the normal BMD and osteopenia. TBA level negatively correlated with BMD in lumbar spine, femoral neck, femoral shaft and ward’s triangle region. The serum TBA level could be employed as a predictor of BMD in T2DM patients. The TBA may be employed as a new therapeutic target for osteoporosis in diabetics (20), which has clinical significance for prevention of osteoporosis in diabetics.

Diabetes mellitus is a complex multifactorial disease (21). In mainland China, diabetes affects 11.2% of adults (22). An important complication of diabetes is osteoporosis. In diabetic, the disorder of glucose and lipid metabolism changes tissue structure and adversely affects bone metabolism, which increased the risk of osteoporosis and fracture (23). Diabetic people suffer a higher risk of fracture compared to the healthy (24, 25), which also correlated with diabetes duration (26–28). Circulating sclerostin level is significantly higher in diabetic persons, which inhibits the function of osteoblasts and bone formation, thus increasing the risk of osteoporosis (29). Osteocalcin is an essential protein for the process of bone formation. Hyperglycemia impairs the function of osteoblasts on synthesis of osteocalcin and then downregulates the osteocalcin level, leading to inhibition of bone formation (30). Increased oxidative stress of platelet mitochondria in T2DM patients interferes with physiological function of bone marrow cells and impairs bone metabolism (31).

TBA, including Chenodeoxycholic acid (CDCA), Tauroursodeoxycholic acid (TUDCA), Deoxycholic acid (DCA), Lithocholic acid (LCA), and 6-alpha-ethyl-chenodeoxycholic acid (6‐ECDCA) and a series of endocrine substance with various physiological functions, is generally synthesized in the liver (32). Multiple pathophysiological mechanisms support the relation between TBA and bone metabolism, including FXR, intestinal flora and Oxidative stress. First, TBA facilitates the differentiation of bone marrow mesenchymal cells into osteoblasts *in vitro* (33, 34). After being treated with DCA *in vitro*, the activity of ALP in bone marrow stromal cells was improved, leading to bone erosion (35). Second, TBA have a positive regulatory effect on osteogenesis by FXR, the principle is CDCA and 6‐ECDCA activates bile acid nuclear receptor FXR. FXR increases the activity of extracellular regulatory protein kinase (ERK) by upregulating runt‐related transcription factor 2 (Runx2), which promotes differentiation of mesenchymal progenitor cells into osteoblast (36). FXR, provokes the expression of ALP, and DNA-binding activity of Runx2, the bone transcription factor (37). Third, intestinal flora regulates bone metabolism through its 7-dehydroxylation producing LCA, a ligand for the vitamin D receptor. Vitamin D regulates the gene coding of bone protein, osteocalcin and receptor activator of nuclear factor-kB ligand (RANKL) (38). Furthermore, LCA affect the formation of osteoblasts and osteoclasts by repressing the expression of calcitonin gene and RANKL gene (16). A dynamic balance exists between diet and intestinal flora-bile acid (39). However, high-fat and cholesterol diet can alter the composition of bile acids in the gut, causing imbalance of intestinal flora and aggravation of bile acid metabolism disorders (40). In addition, oxidative stress plays a role of inhibit osteogenesis by affecting the differentiation, proliferation and apoptosis of osteocytes, and TBA regulates bone metabolism through alleviating oxidative stress (41–44).

Thus far, the clinical evidence for the connection between TBA and bone metabolism is limited. Bile acid malabsorption(BAM) can reduce the absorption of vitamin D and then patients may develop low BMD (45). TUDCA enhance bone tissue regeneration in skull defect models, which can be used as a potential alternative drug for bone regeneration (46).

Following the STROBE guideline (47), we conducted subgroup analyses to make a better use of the data. A retrospective study in China of 2230 healthy persons with aged < 60 and BMI < 25 pointed out that serum TBA was positively correlated with BMD (13). We have different findings, which is that TBA and BMD are negatively related in diabetics with aged < 60 and BMI < 25. Study reported the TBA level was positively correlated with the BMD in postmenopausal healthy population (12). However, we found the TBA level was negatively related with the BMD in postmenopausal diabetics. The above indicated that the pathway of bile acids regulating bone metabolism might be interfered in diabetics. By reference to the mechanism of insulin resistance, we hypothesize that bile acids present a compensatory elevation and have an antagonist effect to osteoporosis. The protective role of TBA in bone metabolism is needed to be explored. In accord with our findings, study reported older age was correlated with decreased BMD and a positive correlation between BMI and BMD (48).

## Limitations

Our findings provided a novel insight into skeletal health in diabetics. Nevertheless, there are some limitations. First, this is a retrospective study based on clinical dataset, which cannot prove the causal relationship between TBA level and bone metabolism. Second, some of participants with osteoporosis has a history of supplementation of calcium and vitamin D, which might bias our findings. Third, although the sample size of this study is high, our evidence might lack of high generalizability and extrapolation due to a single-center study design. Therefore, multi-center prospective studies are needed to offer further identification.

## Conclusion

We observed the negative relevance between TBA and BMD in diabetics, suggesting that a role of bile acids in BMD and bone metabolism among T2DM patients. The circulating TBA level might be employed as an indicator of abnormal bone metabolism.

## Data availability statement

The raw data supporting the conclusions of this article will be made available by the authors, without undue reservation.

## Ethics statement

This study has been reviewed and approved by the ethics committee of TCCH (No. 2021-05-001). As a retrospective study of clinical dataset, this research was exempt from the request of informed consent from subjects.

## Author contributions

ML, QG, and LM designed the study. QW, LD, CX, and YF contributed to data collection. SY, HL, and YG made statistical analysis and manuscript writing. ML, QG, and LM revised the manuscript. All authors have approved the final version.

## Supplementary material

Supplementary Figure 1 Flow-chart of the participants.
