# Supplementary figures and images for: The association between total bile acid and bone mineral density among patients with type 2 diabetes
Source: Front Endocrinol (Lausanne). 2023 Mar 24;14:1153205. doi: 10.3389/fendo.2023.1153205 (PMC10080120; doi:10.3389/fendo.2023.1153205)

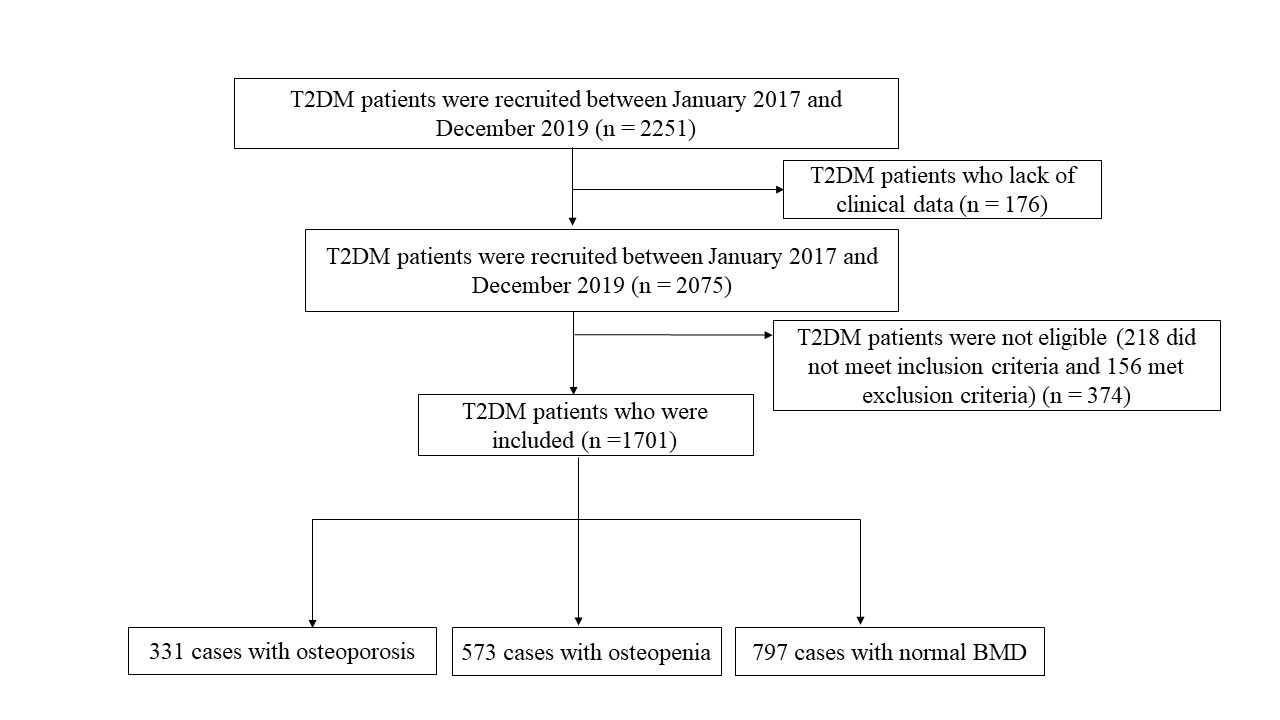

Supplement: Supplementary Figure 1 — Flow-chart of the participants. [file Image_1.tif]
