# Supplementary material for: The association between total bile acid and bone mineral density among patients with type 2 diabetes
Source: Front Endocrinol (Lausanne). 2023 Mar 24;14:1153205. doi: 10.3389/fendo.2023.1153205 (PMC10080120; doi:10.3389/fendo.2023.1153205)
Supplement: Supplementary file 2 [file Table_1.docx]

**Table S1** Multiple linear regression analysis on BMD of total lumbar spine among participants aged < 60 and BMI < 25

| **Indicators** | ***β*  *SE*** | | ***t*** | ***P*** | **95% CI of *β*** | |
| --- | --- | --- | --- | --- | --- | --- |
|  |  |  |  |  | **Lower** | **Upper** |
| Gender (men)  Diabetes duration  TBA  Total bilirubin  Direct bilirubin  Indirect bilirubin | -0.066 | 0.033 | -2.015 | 0.046 | -0.131 | -0.001 |
|  | 0.001 | 0.001 | 0.580 | 0.563 | <0.001 | 0.001 |
|  | -0.022 | 0.006 | -3.639 | <0.001 | -0.034 | -0.010 |
|  | 0.003 | 0.023 | 0.139 | 0.889 | -0.043 | 0.049 |
|  | -0.034 | 0.034 | -1.008 | 0.315 | -0.102 | 0.033 |
|  | 0.011 | 0.021 | 0.522 | 0.603 | -0.031 | 0.054 |

TBA, total bile acid; BMD, bone mineral density; *SE*, standard error; CI, confidence interval; *β*, regression coefficient
