# Supplementary material for: The association between total bile acid and bone mineral density among patients with type 2 diabetes
Source: Front Endocrinol (Lausanne). 2023 Mar 24;14:1153205. doi: 10.3389/fendo.2023.1153205 (PMC10080120; doi:10.3389/fendo.2023.1153205)
Supplement: Supplementary file 3 [file Table_2.docx]

**Table S2** Multiple linear regression analysis on BMD influence factors among men with normal BMI

| **Site** |  | **TBA** |  |
| --- | --- | --- | --- |
|  | ***β*** | **95% CI of *β*** | ***P*** |
| Femoral shaft BMD | -0.016 | (-0.025 ~ -0.007) | 0.001 |
| Total femoral BMD | -0.013 | (-0.020 ~ -0.005) | 0.001 |
| Femoral neck BMD | -0.010 | (-0.016 ~ -0.002) | 0.010 |
| Ward's triangle region BMD | -0.010 | (-0.017 ~ -0.003) | 0.007 |

TBA, total bile acid; BMD, bone mineral density; CI, confidence interval; *β*, regression coefficient; Adjusted for age, diabetes duration, total bilirubin, direct bilirubin, and indirect bilirubin.
