# Supplementary material for: The association between total bile acid and bone mineral density among patients with type 2 diabetes
Source: Front Endocrinol (Lausanne). 2023 Mar 24;14:1153205. doi: 10.3389/fendo.2023.1153205 (PMC10080120; doi:10.3389/fendo.2023.1153205)
Supplement: Supplementary file 4 [file Table_3.docx]

**Table S3** Multiple linear regression of BMD influence factors in postmenopausal women

| **Site** |  | **TBA** |  |
| --- | --- | --- | --- |
|  | ***β*** | **95% CI of *β*** | ***P*** |
| L1 BMD | -0.006 | (-0.011 ~ -0.001) | 0.031 |
| L2 BMD | -0.006 | (-0.011 ~ -0.001) | 0.020 |
| L3 BMD | -0.006 | (-0.012 ~ -0.001) | 0.021 |
| L4 BMD | -0.006 | (-0.011 ~ 0.001) | 0.048 |
| Femoral neck BMD | -0.008 | (-0.015 ~ -0.001) | 0.027 |
| Total lumbar spine BMD | -0.009 | (-0.013 ~ -0.003) | 0.001 |

TBA, total bile acid; BMD, bone mineral density; CI, confidence interval*.β*, regression coefficient; Adjusted for age, diabetes duration, BMI, total bilirubin, direct bilirubin, and indirect bilirubin.
